# Supplementary material for: Multi-channel high-speed flip-chip packaging platform for thin-film lithium niobate photonic circuits
Source: Natl Sci Rev. 2026 Aug 10;13(19):nwag480. doi: 10.1093/nsr/nwag480 (PMC13637627; doi:10.1093/nsr/nwag480)
Supplement: Supplementary file 1 [file nwag480_supplemental_file.pdf]

Supplemental Material for

Multi-channel high-speed flip-chip packaging platform for thin-film lithium niobate photonic circuits

Zhenzheng Wang (汪振政)[1,2], Xiangzhi Xie (谢祥芝)[1,2], Yikun Chen (陈逸堃)[1,2], Yifan Wu (吴怡凡)[1,2], Zeyu Luo (罗泽宇)[1,2], Hanke Feng (冯寒珂)[1,2], Yulong Chen (陈宇龙)[1], Zengyi Xu (徐增熠)[3], Zixi Wang (王梓希)[1,2], Yuansong Zeng(曾元松)[1,2], Mandie Huang(黄曼昳)[1], Ke Zhang (张珂)[4], Yingxia Liu (刘影夏)[5], Cheng Wang (王骋)[1,2,*]

[1]*Department of Electrical Engineering, City University of Hong Kong, Hong Kong 999077, China*

[1]*Department of Electrical Engineering, City University of Hong Kong, Hong Kong 999077, China*

[2]*State Key Laboratory of Terahertz and Millimeter Waves, City University of Hong Kong, Hong Kong 999077, China*

[3]*Key Laboratory for the Information Science of Electromagnetic Waves (MoE), College of Future Information Technology, Fudan University, Shanghai 200433, China*

[4]*Rhinopix Technology Limited, Hangzhou 311100, China*

[5]*Department of Systems Engineering, City University of Hong Kong, Kowloon, Hong Kong 999077, China*

*cwang257@cityu.edu.hk

Note S1. Device fabrication

The fabrication and FC bonding flowchart is shown in Fig. S1. The first four layers of fabrication follow those used in our previous work[1]. Briefly, an ASML UV stepper lithography system was used to pattern the waveguides and optical devices on a thin-film lithium niobate (TFLN) wafer (NANOLN) with a 500-nm-thick device layer. A reactive ion etching (RIE) system was used to perform dry etching, resulting in 250 nm thin-film lithium niobate (TFLN) rib waveguides with a 250 nm slab layer. Through a second lithography step, followed by thermal evaporation and lift-off processes, a 1 µm Cu/Au electrode layer was deposited for the microwave transmission lines. Next, two successive rounds of contact photolithography (using a mask aligner) and thermal evaporation were performed to deposit a 200 nm NiCr layer (for on-chip heaters and 50 Ω loads) and a 1-µm-thick Cu/Au layer (for on-chip routing electrodes), respectively. Subsequently, a 3-µm-thick SiO_2 layer was deposited over the entire chip. Photolithography was then applied using a 7-µm-thick photoresist (AZ 2070) to cover all areas except the bonding pads. The SiO_2 above the bonding pads was fully dry etched to expose them, followed by thermal evaporation of a final 2-µm-thick Cu/Au layer. This step formed the vias and increased the

thickness of the bonding pads to a total of 3 μm, sufficient for effective reaction with the solder balls during the subsequent flip-chip (FC) bonding process. Next, Sn solder balls with a diameter of 250 μm were planted on the pads of the printed circuit board (PCB) made of Rogers-series low-loss materials. Notably, our initial approach employed stencil-based solder paste printing to form solder balls. With this method, the minimum achievable bonding pad diameter was 250 μm, and both the PCB and the chip were designed accordingly for this pad size. Although we have since switched to machine-based solder-ball placement—capable of producing solder-ball diameters as small as several tens of micrometers—the sizes of the bonding pads and the solder balls have remained unchanged. Subsequently, precise FC bonding was performed using a high-accuracy surface-mount technology (SMT) pick-and-place machine. During this process, the PCB was securely mounted on the work stage, while the chip was picked up from its backside using a vacuum nozzle enabling free motion along the x, y, and z axes. Alignment was achieved with the machine's vision system (nominal accuracy ±0.5 μm), and the practical bonding alignment accuracy was approximately ±5 μm. After alignment, the assembly was preheated and approximately 1 N of pressure was applied to preliminarily fix the relative positions of the PCB and the chip. Additionally, vacuum reflow soldering with a maximum temperature of 260 °C was performed to minimize oxidation of the solder and bonding pads during the bonding process, thereby ensuring excellent solder-joint quality and yielding the PCB assembly (PCBA) module. Finally, to further enhance the bonding strength of the optical chip to the PCB, we applied adhesive to its four corners.

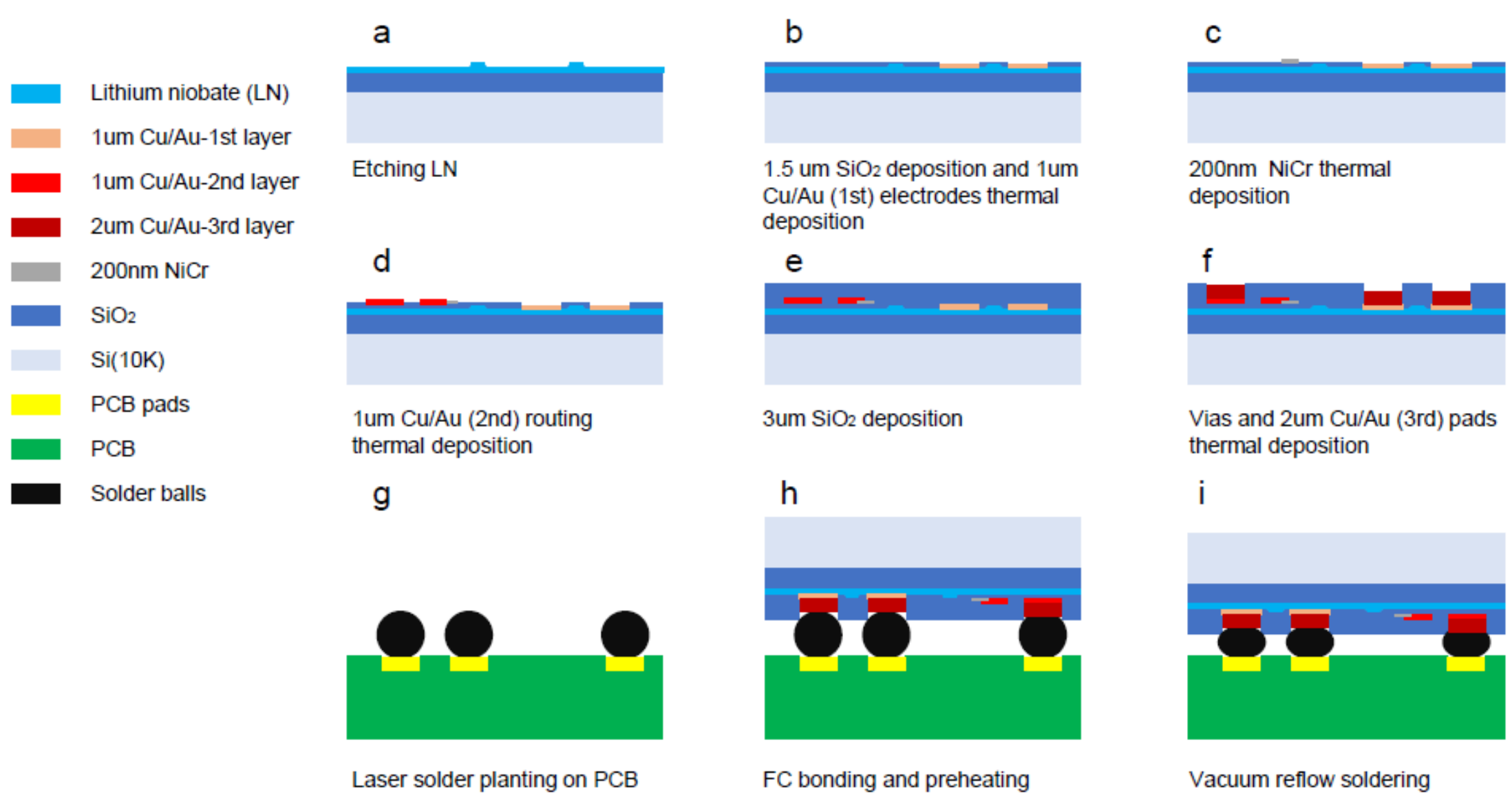

Figure S1. Process flowchart of the photonic chip fabrication and FC bonding.

Note S2. Electro-optic characterization of individual modulators

A continuous-wave (CW) laser (Santec TSL-550) was employed as the light source in the optical characterizations, with its center wavelength set to 1550 nm and tunable over the telecom C and L bands. A three-paddle fiber polarization controller (FPC) was used to ensure excitation of the fundamental transverse-electric (TE) mode. Light was butt-coupled into and out of the chip using lensed fibers.

To measure the half-wave voltage (V_π), a 500-kHz triangular-wave voltage signal with a peak-to-peak voltage of 4 V was applied to AM 3-1 via the PCBA. Input light was sent to port 'Add 3-1' to bypass other on-chip components, while output port 'O1' was monitored using a low-noise PD and a real-time oscilloscope (Rohde & Schwarz RTP series, 13 GHz bandwidth).

The electro-optic (EO) S21 parameters of the AM 3-1 were measured using a high-speed photodetector (PD; Finisar XPDV21X0RA, 50 GHz bandwidth) and a vector network analyzer (VNA; Keysight E5080B, operating from 9 kHz to 53 GHz).

Note S3. Characterization of the 2×8 optical switch

To measure the transmission spectra under various switching states, light was first coupled into the device through the 'input' port and out through the 'O1' port. By continuously electro-optically tuning the first two stages of modulators (AM 1-1 and AM 2-1), the optical power at the 'O1' output port was maximized, thereby optimizing the power delivered to the third-stage modulator (AM 3-1). Subsequently, AM 3-1 was electro-optically tuned to maximize the output power—initially at 'O1' and then at 'O2'—while the output spectra at both ports were recorded simultaneously. The same procedure was repeated for the output port pairs 'O3'/'O4', 'O5'/'O6', and 'O7'/'O8'.

To measure the rise and fall times, the first two stages of modulators were first adjusted to maximize the optical power routed into AM 3-1, after which AM 3-1 was tuned to its quadrature bias point. Next, a 1 GHz periodic rectangular wave was generated using a high-speed arbitrary waveform generator (AWG; Keysight M8196, 32 GHz bandwidth), and amplified by a low-noise electrical amplifier (EA; SHF S807C, 70 kHz–55 GHz), before being applied to the EO electrode of AM 3-1. The output optical signal was detected using the 50 GHz PD and recorded on the real-time oscilloscope. The peak-to-peak voltage (Vpp) of the rectangular wave from the AWG was adjusted to achieve full switching between the band-pass and all-stop states.

Note S4. Characterization of the EO comb-based photonic transmitter

The output port ‘O1’ in Fig. 4a served as a monitoring port for the EO comb source only, while the output port ‘O2’ was utilized to characterize the performance of the full EO comb-based photonic transmitter.

To drive the EO comb, a 50 GHz RF signal, generated by a microwave source, was first split by a 3 dB electrical power splitter. One path was phase-shifted, amplified by an electrical amplifier (EA 1, model TMPA-040060-3036-19), isolated, and directed to the PM, while the other was amplified by a second electrical amplifier (EA 2, model TLLA40G50G-40-28), appropriately attenuated, and then applied to AM 1. When only driving the PM (Fig. 4b), the second path was bypassed and all RF power was applied to the PM. Output comb spectra were monitored using an optical spectrum analyzer (OSA). To achieve flat-top EO comb generation, AM 1 was first thermally tuned to its quadrature bias point. Subsequently, the phase shifter was adjusted to control the phase difference between the RF signals applied to AM 1 and the PM, while the attenuator was simultaneously tuned to optimize the RF power delivered to AM 1, until optimal spectral flatness of the comb was obtained.

For eye diagram tests, erbium-doped fiber amplifiers (EDFA) were used both before and after the chip to ensure sufficient optical power into the 50 GHz PD. AM 2 was thermally tuned to its quadrature bias point. A high-speed AWG generated a random on-off keying (OOK) signal, which was subsequently amplified by a low-noise EA 3 (SHF S807C) and applied to AM 2 via the PCBA. Finally, eye diagrams were acquired using the real-time oscilloscope.

Note S5. The effect of the large PCB metal plate

As shown in Fig. 2b, a large metal plate is present in the PCB bonding area, and after FC bonding, the gap between the chip and the PCB is approximately 100 μm. To analyze the effect of the large PCB metal plate on the modulator, we simulated a 400-μm-long ground-signal-ground (GSG) transmission line on the TFLN chip using HFSS and extracted its S-parameters. A large metal plate was then placed 100 μm above the chip, and the S-parameters were re-extracted. To suppress unwanted resonances in the simulation, the impedances of the two lumped ports were matched to the characteristic impedance of the transmission line, resulting in the small EE S11 and EE S22 values in these simulations. By comparing the S-parameters obtained before and after the introduction of the metal plate, the impact of the large-area PCB metal plate was assessed. As shown in Fig. S2, the extracted S-parameters show negligible changes, demonstrating that the PCB metal plate has minimal influence on the modulator performance for the current PCB-chip gap.

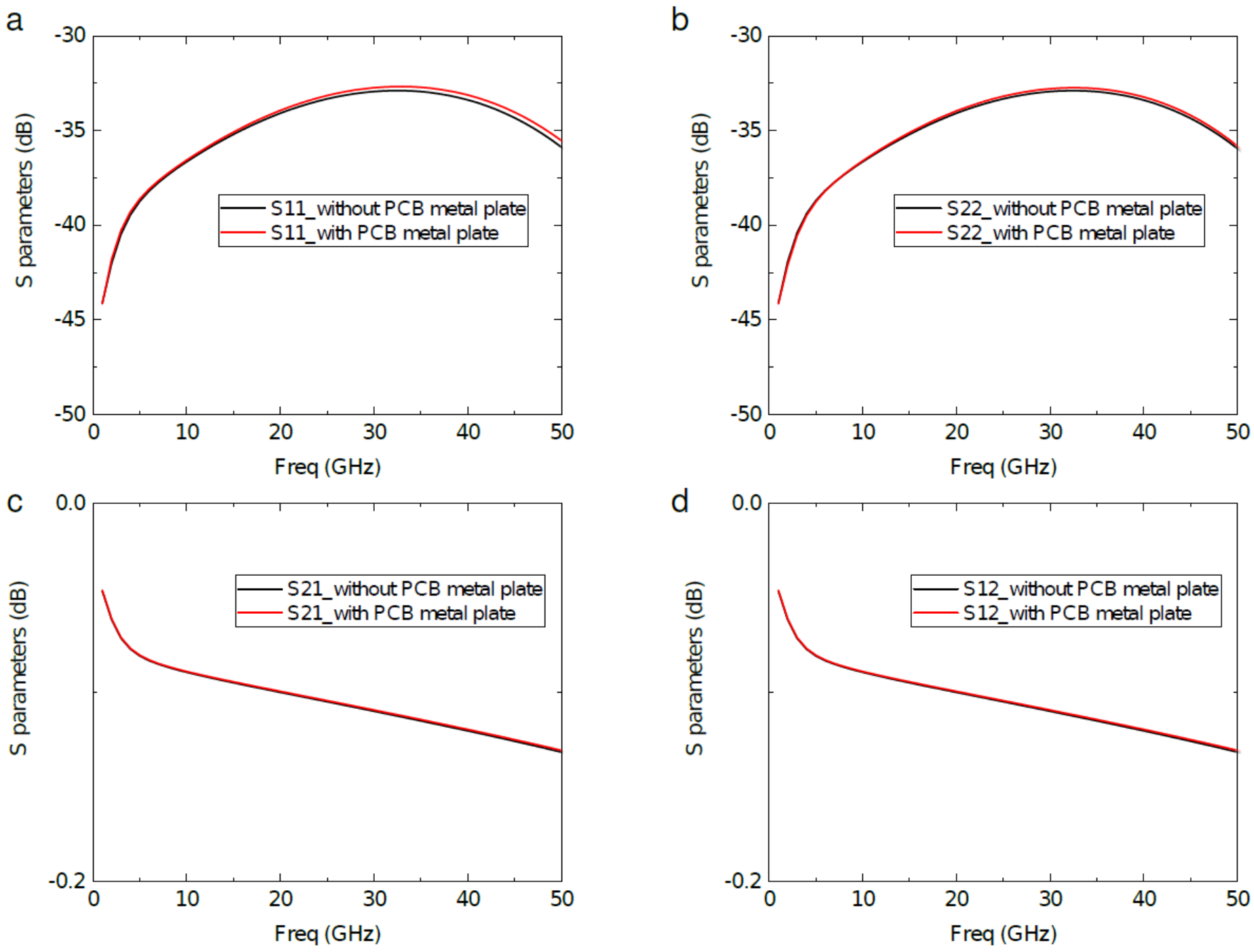

Figure S2. Comparison of simulated S-parameters for a 400 μm long GSG transmission line with (red curve) and without (black curve) a PCB metal plate at a distance of 100 μm from the transmission line. (a) S11, (b) S22, (c) S21, (d) S12.

Supplementary References

1. Wang Z, Chen Z, Feng H *et al.* Integrated lithium niobate Vernier micro-ring filter with wide and fast tuning capabilities. *Opt Express* 2024; **32**(25): 44272–9.
